# Continuous improvement of a bioengineering CURE: Preparing students for a changing world

**DOI:** 10.1002/bmb.21656

**Published:** 2022-08-05

**Authors:** Chandrani Mishra, Lauren Novak, Coleen Riley, Ikenna Okekeogbu, Gillian Smith, Emma Brace, Emily Kerstiens, Kari Clase

**Affiliations:** ^1^ Department of Agricultural and Biological Engineering Purdue University West Lafayette Indiana USA; ^2^ Biological Engineering Purdue University System West Lafayette Indiana USA

**Keywords:** ABET, assessment, CURE, undergraduate research

## Abstract

Based on recent education reform guidelines to prepare professionals who are able to handle new technological, economic, social, and environmental challenges, pedagogical modifications are deemed necessary by the educators. Specifically, in biology, the rapid changes in the content and biological products demand changes in the curriculum. We aim to address this current need by providing an example of a course that was redesigned to meet the current trends of biological engineering education. In this course‐based undergraduate research experience (CURE), learning objectives and possible outcomes were developed and assessment mapping was performed to align the course objectives with the Accreditation Board for Engineering and Technology (ABET) recommendations. A description of how one can assess authentic inquiry courses while adhering to the recommendations are discussed. For example, in this particular course, students completed weekly reflection assignments, maintained lab notebooks that were graded every week, presented their research to their peers at the end of the semester, and submitted a final paper to be graded. “Holistic” engineering is crucial for the all‐around development of a 21st century engineer. Altering the traditional lecturing with more hands‐on learning is crucial for the development of professional and communication skills of students. Such alterations could lead to the production of well‐rounded life‐long learners to serve the upcoming world.

## Graphical abstract

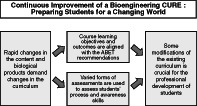

## 1 BACKGROUND

The latest challenges in the 21st century with regard to engineering education are no longer limited to training technical experts in the field but also to prepare professionals able to handle new technological, economic, social, and environmental challenges.^1^ In order to achieve that, courses including all science, technical and engineering ones should be designed in a way to develop professional competencies among students such as the ability to use modern engineering techniques, communication, ability to function in a team, and problem‐solving skills.^2^ A long‐term desire for change in the traditional nature of engineering education led to the origin of the term “holistic engineering”, rightly coined by Joseph Bordogna, a University professor and former deputy director of the National Science Foundation.^1^ Through this term, he referred to the need for incorporating cross‐disciplinary and whole system thinking approaches in engineering curriculum. Holistic engineering is envisioned as a means to meet the challenges of the 21st century by introducing different types of professional practices in engineering education.^1^ Similarly, other studies have identified the need for the development of competency for innovation, the ability to work in a global setting, and the ability to encounter new challenges among the new graduates.^3^, ^4^, ^5^, ^6^

In particular, students in biological engineering and other fields that work with biomaterials are expected to enter the workforce with up‐to‐date skills on the newest advances in biology and biological products. The rapid changes in biologic operations and products demand curriculum content changes and this demands changes in pedagogy.^7^ Pedagogical changes such as how we teach, coupled with changes in how we assess and the development of new assessments that are aligned with the content changes and competency demands are essential. ABET was established in order to formalize these demands for the development of professional competencies in engineering curricula.^8^ For promoting and advancing engineering education, ABET incorporated six professional skills (three process skills and three awareness skills) in addition to the existing hard skills (such as technical and analytical) in engineering education curriculum as follows:

- an ability to function on multi‐disciplinary teams
- an understanding of professional and ethical responsibility
- an ability to communicate effectively
- the broad education necessary to understand the impact of engineering solutions in a global, economic, environmental, and societal context
- a recognition of the need for, and an ability to engage in lifelong learning
- a knowledge of contemporary issues

Among several benefits of a global accreditation model, uniformity, and systematic application of accreditation criteria and assessment is very crucial.^1^ To help educators re‐design courses to align with the accreditation policies, ABET outlined student outcomes that comprise all the engineering and professional skills that students should acquire through any engineering course they take.

Several institutions are advocating various teaching practices to support the development of professional skills among recent graduates.^9^, ^10^ However, more examples of how current engineering courses can be redesigned and assessed to meet the demands of the development of professional competency is essential. In this paper, we aim to address this current need by providing an example of a course that was redesigned to meet the current trends of biological engineering education. We also elaborate on how we mapped our assessment strategies to the course learning objectives aligned with the ABET recommendations.

One other aspect of this course is the involvement of the undergraduate peer leaders. Due to the positive impact of peer influence on student success, higher education leaders have started involving undergraduate students in peer leadership roles to benefit the students.^11^ Studies suggest that peer leadership experience leads to the development of skills such as excellent oral communication, critical thinking, teamwork, problem solving, and leadership, all of the same that are desirable among higher educators and employers.^11^, ^12^ For example, in a podcast discussion regarding critical skills for the future success of businesses, speakers highlighted essential skills such as technology and human skills including the ability to adapt, collaborate, and communicate.^13^

## 2 METHODS/COURSE DESIGN

The course used as an example in this study is a course‐based undergraduate research experience (CURE) and a core course in a department of Agricultural and Biological Engineering that is open to Biology majors, taught at a midwestern public research university. More broadly, the course is a part of the Howard Hughes Medical Institute's (HHMI) Science Education Alliance Phage Hunters Advancing Genomic and Evolutionary Science (SEA‐PHAGES) project (http://www.hhmi.org/grants/sea/). In this course, students engage in hands‐on discovery as scientists with the ultimate objective of contributing new mycobacteriophage (virus that infect mycobacteria) genomes to the public databases. Students isolate bacteriophage from soil samples, purify and amplify them, extract their DNA, and finally characterize them via electron microscopy and gel electrophoresis in the fall semester. During winter break, DNA samples are sent to an external site for genome sequencing. All participants share their discoveries, ideas, and challenges via the HHMI Science Education Alliance wiki.

The objective of this course is to introduce the student to the step‐by‐step process of scientific discovery while developing techniques commonly used in biotechnology research in both academic and industrial settings. Students maintain a scientific notebook, learn to apply experimental design, develop critical thinking skills in the critique of journal articles, and use computer databases. More specifically, this course focuses on current laboratory techniques used to isolate, manipulate, and identify biological molecules such as nucleic acids and proteins. Basic laboratory techniques (pipetting, solution making, buffer preparation, good safety techniques), aseptic techniques, and compliance procedures are also discussed.

This is an example of course‐based undergraduate research experiences (CUREs). Such CUREs can help engage a large number of students in a research experience and address valuable learning outcomes that help them learn authentic scientific thinking and practice. Additionally, this course provides students that have completed the research experience in ABE 22600 and ABE 22700 an opportunity to continue their professional development by serving as peer leaders in the classroom. As part of the model for implementing the course‐based undergraduate research experience, upperclassmen that successfully completed those courses serve as peer leaders in the classroom. Peer leaders attend the course section along with the currently enrolled students and build competencies in leadership by guiding their team on their research project throughout the semester. More specifically, peer leaders:

o mentor peers in the use of scientific practices

o guide peers through the process of discovery including iteration as their team isolates and characterizes a unique mycobacteriophage and designs a phage synthetic biology research project

o mentor peers in collaboration by guiding their efforts to work productively together on a team and communicate the results of their project

The objective of our study was to transform an existing CURE for undergraduate students at a large land‐grant institution. Learning outcomes and objectives of this course were developed and assessment mapping was performed to align the course objectives with ABET recommendations. Table 1 represents specific course activities in the course that helps in the development of professional skills identified by ABET.

**TABLE 1:** Representation of how course activities help to develop professional skills identified by ABET in this course.

a. Ability to function on multi‐disciplinary teams	b. Understanding of professional and ethical responsibility	c. Ability to communicate effectively	d. Understand the impact of engineering solutions in a global, economic, environmental, and societal context	e. Ability to engage in lifelong learning	f. Knowledge of contemporary issues
Students work in pairs and have to communicate their work beyond the classroom	Each student is responsible for the project that they share with their partner	Students participate in an in‐class presentation	Students participate in reflection activities where they have to contemplate the overall goal of the project and impact of their project on a global context	Students are engaged in hands‐on learning which helps to build their professional, scientific identity that helps to engage in lifelong learning	Students participate in reflection activities which often includes readings from recent discoveries in the field to keep them updated with the knowledge of contemporary issues

During Fall 2020, the course was adapted because of COVID‐19 social distancing requirements. Due to in‐person capacity limits, less than half of the students were permitted in the teaching lab per course section. To ensure that the CURE learning outcomes were still addressed with the modified course structure, the students attended lab and virtually attended a lab group meeting on alternating weeks. When the students were not attending these meetings, they were learning content from supplemental learning modules and applying their knowledge in their lab notebooks, reflections, and a synthetic biology final project.

## 3 RESULTS

### 3.1 Course learning objectives mapping to ABET student outcomes

#### 3.1.1 ABET student outcome 1

An ability to identify, formulate, and solve complex engineering problems by applying principles of engineering, science, and mathematics.

**Table:** 

**Course learning outcome**	**Justification of mapping the course learning outcome to ABET student outcome 1**
Students will be involved in the use of scientific practices.	The student will be able to explain the experimental basis of techniques used, indicating the significance of the work, presenting, calculating, and discussing the data, and drawing conclusions. The student will be able to identify and define the basic terms within the field of biotechnology. The student will acquire basic research skills. Such as the ability to perform techniques currently used in cell, molecular, and microbiology, while understanding the rationale behind the specific approaches. The student will gain experience in dissecting and extracting pertinent information from scientific journal articles. The students will propose solutions to troubleshoot and re‐design or improve failed experiments.
**How is it assessed**	Graded lab notebooks and reflection assignments due every week.

##### Sample students' responses

*Reflection question*: After two rounds of serial dilution, a student group in the ABE 226 class observed 55 plaques on a plate from a culture infected with 0.01 ml of a 10^−4^ dilution. What is the titer in pfu/ml of the phage lysate? Make sure to include your mathematical work. How do you think equations represent what you are finding?

*Student response*: 55 plaques formed from the 10 μl plated, then the μl is converted to ml (10^3^), then the dilution factor of the plate is taken into account (10^4^) which generates this equation to determine how many pfu are in each ml. Titer = 55/10 μl × 10^3^ × 10^4^ = 5.5 × 10^7^ pfu/ml.

#### 3.1.2 ABET student outcome 2

An ability to apply engineering design to produce solutions that meet specified needs with consideration of public health, safety, and welfare, as well as global, cultural, social, environmental, and economic factors.

**Table:** 

**Course learning outcome**	**Justification of mapping the course learning outcome to ABET student outcome 2**
Students will be involved in broadly relevant or important work.	The students will isolate new phages by the end of the semester which has implications for public health as phages could provide a solution to antibiotic resistance. The student will compare the structure and properties of the phage to other phages. The student will share the information gathered with other students in the class and the scientific community outside the class.
**How is it assessed**	Graded final paper and final presentation due at the end of the semester. Graded lab notebooks and reflection assignments due every week.

##### Sample students' responses

*Reflection question*: If two phages look very similar by electron microscopy, would you predict that they will have similar genomes? Why or why not?

*Student response*: One could predict that they might have similar genomes. This is because genes often control the phenotype of the phage's morphology. If they have similar structures, they may have similar genome sequences that code for similar proteins. At the same time, phage genomes can be widely varied despite sharing similar structures because of many other different properties such as their life cycle or the bacteria they infect. These different functions can show varied genomes in phages that look similar.

#### 3.1.3 ABET student outcome 3

An ability to communicate effectively with a range of audiences

**Table:** 

**Course learning outcome**	**Justification of mapping the course learning outcome to ABET student outcome 3**
Students will be involved in broadly relevant or important work. Students will be involved in collaboration.	The student will share the information gathered with others in the scientific community. Students create a phage page in the PhagesDB database (https://phagesdb.org/institutions/PURD/) and include information such as the phage's electron microscopy image, the soil sample information (GPS coordinates of where the sample was found), and a photo of what the plaque looks like. This database is used by the wider scientific community.
How is it assessed	Graded final paper and final presentation due at the end of the semester. Ability to communicate clearly in graded lab notebooks due every week.

#### 3.1.4 ABET student outcome 4

An ability to recognize ethical and professional responsibilities in engineering situations and make informed judgments, which must consider the impact of engineering solutions in global, economic, environmental, and societal contexts.

**Table:** 

**Course learning outcome**	**Justification of mapping the course learning outcome to ABET student outcome 4**
Students will be involved in the use of scientific practices.	Students will be required to put on lab coats and safety glasses. Students will be encouraged to establish ethics in naming their phages. Students will be advised to dispose of biohazard materials responsibly. Students will be discouraged from plagiarizing and/or falsifying or manipulating data.
**How is it assessed**	Timely submissions of lab notebooks and reflection assignments due every week. A portion of the students' laboratory performance grade was based on their professionalism.

#### 3.1.5 ABET student outcome 5

An ability to function effectively on a team whose members together provide leadership, create a collaborative and inclusive environment, establish goals, plan tasks, and meet objectives.

**Table:** 

**Course learning outcome**	**Justification of mapping the course learning outcome to ABET student outcome 5**
Students will be involved in collaboration.	The student will work on a team and communicate their results. Students will be responsible for collaborating with others, dividing tasks, managing project materials and milestones, and making progress such that they meet all the goals by the end of the term.
**How is it assessed**	Graded lab notebooks due every week. Graded final paper and final presentation due at the end of the semester.

##### Sample from students' lab notebook

Figure 1.

**FIGURE 1:**
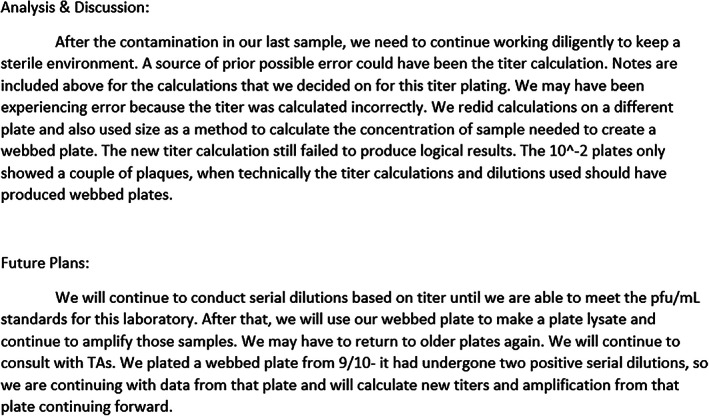
Snapshot of students' lab notebook showing how they plan and work effectively as a team.

#### 3.1.6 ABET student outcome 6

An ability to develop and conduct appropriate experimentation, analyze and interpret data, and use engineering judgment to draw conclusions.

**Table:** 

**Course learning outcome**	**Justification of mapping the course learning outcome to ABET student outcome 6**
Students will be involved in the process of discovery. Students will be involved in iteration.	The student will isolate and characterize a unique mycobacteriophage. The student will be able to navigate uncertainty. The student will troubleshoot and conduct research to contribute new knowledge about the unique phage. The student will analyze data from the previous week and use them to make informed decisions on experiments for upcoming weeks.
**How is it assessed**	Graded lab notebooks of students due every week. Graded reflection assignments also due every week.

##### Sample students' responses

*Reflection question*: Reflect on your experience with failure as a part of authentic research and consider the following as you reflect: a. What have you learned from your failure? Provide details.

*Student response*: During some of our direct isolations, we had a lot of contamination. However, for example, 3 weeks, we had two soil samples and did direct isolation on them for ABE 226 Lab Section: 11:30–1:30 4 a total of four plates, but only one plate out of all the samples was contaminated. There was much confusion on why this was contaminated. We discussed with the peer TA and she helped us with our aseptic technique, looking over our shoulder to ensure we were doing it correctly. Another failure we have had is not being able to find phages. Most of our samples come back entirely blank, with no plaques or contamination. We hope to find phages in our enriched isolation sample. We have learned that it is not easy to find something that is 1 million times, or even more, smaller than you.

#### 3.1.7 ABET student outcome 7

An ability to acquire and apply new knowledge as needed, using appropriate learning strategies.

**Table:** 

**Course learning outcome**	**Justification of mapping the course learning outcome to ABET student outcome 7**
Students will be involved in iteration.	The student will troubleshoot and conduct research to contribute new knowledge about the unique phage.
**How is it assessed**	Graded reflection assignments asking them to reflect on their failures and troubleshooting strategies to get success. Students also report their failures or use of alternative strategies to conduct their experiments in their lab notebooks.

##### Sample from students' lab notebook

Figure 2.

**FIGURE 2:**
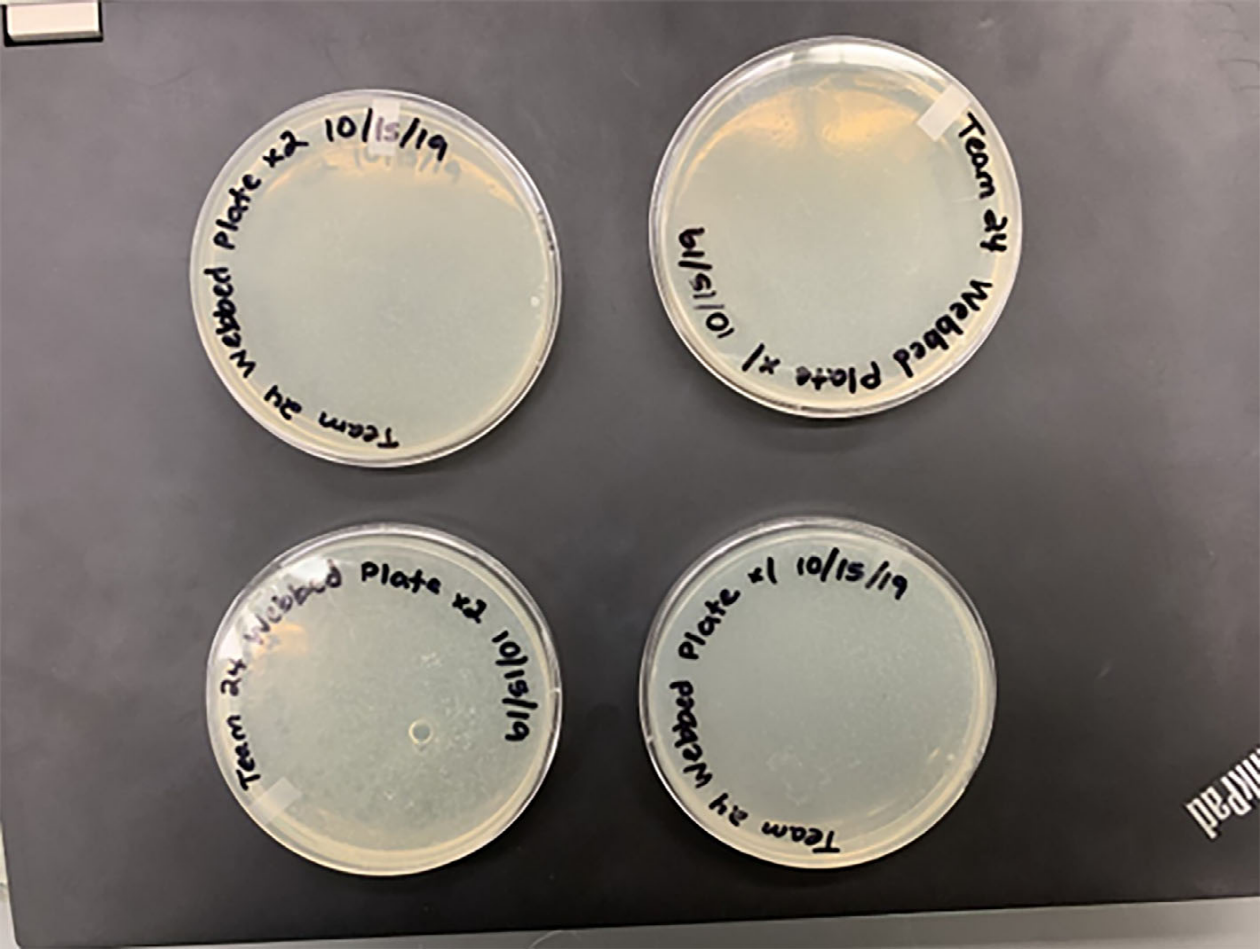
A snapshot of a student's lab notebook showing their ability to acquire and apply new knowledge as needed using appropriate learning strategies.

### 3.2 Assessments overview

In courses like the one described above which, unlike a traditional course, is more open‐ended and involves students in authentic inquiry, instructors often struggle to assess the students' learning outcomes. Therefore, we provide a description of how we can assess authentic inquiry courses as well as meet ABET recommendations. Students completed weekly reflection assignments, maintained lab notebooks that were reviewed and graded every week, presented their research to their peers at the end of the semester, and submitted a final paper to be graded. In addition to these, a portion of students' grade was also based on their professional performance in class. Examples from student reflections and lab notebooks are provided in the Appendix S1 to illustrate how varied forms of assessments are used to evaluate student outcomes. Described below are the varied forms of assessments that are used in this course.

- *Graded lab notebooks*: Because students' work in this course is real research, the output of students' assignments and work will likely be used by the broader scientific community for years to come. For this reason, documentation of their work and the data collected is critical. Students maintain an electronic laboratory notebook using Google Docs and are graded on the completeness, organization, accuracy, ease of use by outside readers, and adherence to basic research documentation protocols. Lab notebooks were graded every week.
- *Reflection assignments and assigned readings*: Readings are assigned to provide more information and background on the concepts applied in the research laboratory. There are oral discussions over the readings and occasional writing activities. Discussion questions are also included in the reflections. Reflections are completed weekly and submitted electronically to provide updates of laboratory progress, troubles encountered, and the application of foundational scientific content knowledge related to the research project. Sample reflections questions include:
  - Think of an experiment to perform if you continue to obtain more than one morphology in your phage preparations (e.g., some plaques are large and clear, and some are much smaller and cloudy).
  - Describe the aseptic techniques we use in class and note at least three critical factors for practicing aseptic technique. Why is it important to use aseptic techniques?
  - What issues or challenges did you face this week? What did you do to overcome those challenges?
- *Final paper and final presentation*: A final research paper and a class presentation are completed by students at the conclusion of the semester to both summarize research findings and outline potential future areas of research. Readings from the scientific literature are assigned throughout the semester to help provide a foundation for the literature review. Some of the prompts to help students prepare the presentation include:
  - Objectives of your semester research
  - Images and explanation of your experimental data
  - Results and conclusions; What would be your future work if you could continue?
  - Troubleshooting: What problems did you encounter? How did you overcome them? Did you have critical decision points in your project? How did you decide your next steps? What would you improve if you could go back? What advice do you have for next year's phage hunters?
- *Laboratory performance*: A portion of students' grade (10%) also depends on laboratory performance including, but not limited to, any of the following:
  - Attendance and punctuality
  - Being unprepared such as forgetting to review their lab manual
  - Leaving the laboratory before completing the exercise
  - Failing to clean up after an experiment
  - Violating safety regulations
  - Conducting oneself unprofessionally

- Lack of effort and forethought in experimental design

### 3.3 Students' evaluation of the course

**FIGURE 3:**
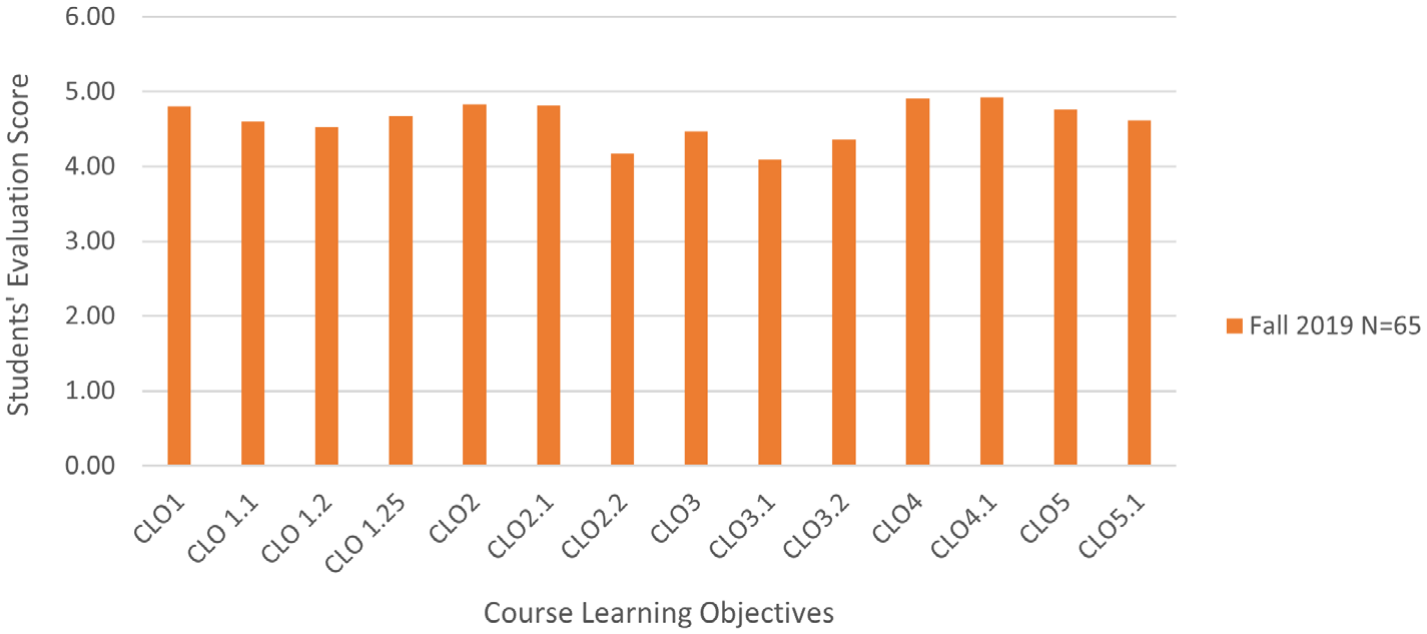
Students' overall evaluations of the course

Students were asked to respond to a course evaluation survey specifically on the extent to which the course learning objectives were met. On a scale of 6, students rated each of the course learning objectives as 4 or higher (Figure 3). Course learning objective 4, according to which students were able to collaborate with others was rated the highest followed by the course learning objective 2, according to which students had the opportunity to involve in the process of discovery.

## 4 DISCUSSION

Innovative teaching approaches that uplift the standards of the curriculum contribute overall to elevation of students' performance, interest, and classroom environment. In addition, assessing a CURE or any other traditional courses with forms of assessments beyond traditional quizzes and exams has always been challenging.^14^, ^15^ Furthermore, assessments have been rarely discussed for lab‐based courses and more rarely have been linked to the learning goals.^15^ In this regard, using lab notebooks and reflections as alternative yet effective forms of assessment are helpful. Appendix S1 provides concrete examples of how a particular assessment was used to assess recommended student outcomes. A detailed analysis of how students performed in the assessments beyond the semester of focus for this study, however, remains to be explored. The example from our course is intended to provide educators with concrete examples of how an authentic lab‐based biological engineering course can be modified to meet ABET outcomes and overall prepare students for professional careers in tomorrow's world. It can also be adapted for other authentic lab‐based science and engineering courses with similar learning goals. Student feedback and collaboration with the peer leadership team enables continuous improvement of the course each year. Through this process of reflection and innovation, the desired CURE learning outcomes for the course are achieved—even in the midst of the ongoing global pandemic.

## 5 CONCLUSIONS

Re‐designing the courses to meet the current trends of biological engineering education provides opportunities for students to develop necessary process and awareness skills in addition to a strong knowledge of technology. An approach towards a “holistic” engineering is crucial for an all‐round development of a 21st century engineer. Some modifications of the existing curriculum could help with the incorporation of a holistic engineering approach. The more the students are provided with opportunities to use their process and awareness skills, the more they are prepared for the world ahead. Additionally, altering the traditional lecturing with more hands‐on learning is crucial for the development of professional and communication skills of students. Such alterations could lead to the production of well‐rounded life‐long learners to serve the upcoming world.

## Supporting information


**APPENDIX S1**: Examples from student reflections and lab notebooks.
