# Supplementary material for: Continuous improvement of a bioengineering CURE: Preparing students for a changing world
Source: Biochem Mol Biol Educ. 2022 Aug 5;50(5):510–8. doi: 10.1002/bmb.21656 (PMC9804359; doi:10.1002/bmb.21656)

**Supplemental File 1: Examples From Student Reflections And Lab Notebooks**

**REFLECTION EVIDENCE**

**ABET Student Outcome 1 - An ability to identify, formulate, and solve complex engineering problems by applying principles of engineering, science, and mathematics**

| **Course learning outcome**  Students will be involved in the use of scientific practices. | **Justification of mapping the course learning outcome to ABET student outcome 1**   - The student will be able to explain the experimental basis of techniques used, indicating the significance of the work, presenting, calculating, and discussing the data, and drawing conclusions. - The student will be able to identify and define the basic terms within the field of biotechnology - The student will acquire basic research skills. such as the ability to perform techniques currently used in cell, molecular, and microbiology, while understanding the rationale behind the specific approaches. - The student will gain experience in dissecting and extracting pertinent information from scientific journal articles. - The students will propose solutions to troubleshoot and re-design or improve failed experiments. |
| --- | --- |
| **How is it assessed:** | Graded Lab Notebooks and Reflection Assignments due every week. |

Q: After two rounds of serial dilution, a student group in the ABE 226 class observed 55 plaques on a plate from a culture infected with 0.01 ml of a 10-4 dilution. What is the titer in pfu/ml of the phage lysate? Make sure to include your mathematical work. How do you think equations represent what you are finding?

Sample student response: *55 plaques formed from the 10uL plated, then the uL is converted to mL (10^3), then the dilution factor of the plate is taken into account (10^4) which generates this equation to determine how many pfu are in each mL. Titer= 55/10uL x 10^3 x10^4= 5.5 x10^7pfu/mL.*

Q: See the pictures to answer the questions below:

**
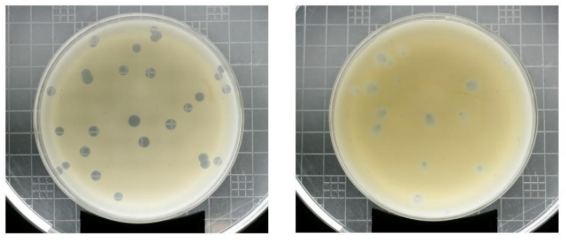
**

a. Which phage (left or right plate) is likely to be lytic and which is likely to be temperate? Provide a rationale to support your answer.

Sample student response: *The left plate is likely to be lytic because it demonstrates the classic, perfectly clear and circular plaques. The right plate is likely the temperate phage because it has some opaqueness and also has a “bulls eye” pattern.*

b. What experiment could you design to test your guess?

Sample student response: *Similar to a spot test to determine if a phage is present in a sample, an experiment could be designed to determine if the phage is a temperate phage or a lytic phage based on the presence of lysogens. Because temperate phages have plaques that are turbid, lysogens are still present in the center of the plaques. As opposed to lytic phages and plaques, there are not lysogens present in the plaques, as they are all killed.*

c. What data would you expect from your experiment if it is a lytic phage?

Sample student response: *If it is a lytic phage, no lysogens would be present in the sample.*

d. What data would you expect if it is a temperate phage?

Sample student response: *If it is a temperate phage, then lysogens would be present in the sample.*

______________________________________________________________________________

**ABET Students Outcome 2 - An ability to apply engineering design to produce solutions that meet specified needs with consideration of public health, safety, and welfare, as well as global, cultural, social, environmental, and economic factors.**

| **Course learning outcome**  Students will be involved in broadly relevant or important work. | **Justification of mapping the course learning outcome to ABET student outcome 2**   - The students will isolate new phages by the end of the semester which has implications for public health as phages could provide a solution to antibiotic resistance - The student will compare the structure and properties of the phage to other phages - The student will share the information gathered with other students in the class and the scientific community outside the class |
| --- | --- |
| **How is it assessed:** | Graded Final Paper and Final Presentation due at the end of the semester. Graded Lab Notebooks and Reflection Assignments due every week. |

Q: If students isolate and sequence the DNA of phages from different sites nationwide, what

kinds of research questions can be answered with the data they collect, assuming they

record all the requested sampling information?

Sample student response: *Assuming all of the students record all the requested sampling information, dozens of research questions can be answered from this data. For example, many research questions relating to the types of bacteriophages discovered and their respective locations can be answered, such as: does the respective collection location correspond with the distinguishable types of mycobacteriophages present in the soil? In addition, are certain mycobacteriophages present in different parts of the country? Finally, are mycobacteriophages present more readily in warmer or cooler and/or wet or dry conditions?*

**Q: How did the EM help with the characterization of phage, specifically the type of phage that we are working with this semester?**

Sample student response: *Electron microscopy ultimately allowed us to capture an image of our phage and observe its structure such as capsid length, capsid shape, and tail length. These characteristics can indicate the type of phage we are working with because the morphotype of the phage often dictates what family it belongs to. For example, our phage had a long tail and long capsid which indicated it may be part of the Syphoviridae family.*

**Q: If two phages look very similar by electron microscopy, would you predict that they will have similar genomes? Why or why not?**

Sample student response: *One could predict that they might have similar genomes. This is because genes often control the phenotype of the phage's morphology. If they have similar structures, they may have similar genome sequences that code for similar proteins. At the same time, phage genomes can be widely varied despite sharing similar structures because of many other different properties such as their life cycle or the bacteria they infect. These different functions can show varied genomes in phages that look similar.*

_____________________________________________________________________________

**ABET Student Outcome 6 - an ability to develop and conduct appropriate experimentation, analyze and interpret data, and use engineering judgment to draw conclusions**

| **Course learning outcome**   - Students will be involved in the process of discovery - Students will be involved in iteration | **Justification of mapping the course learning outcome to ABET student outcome 6**   - The student will isolate and characterize a unique mycobacteriophage. - The student will be able to navigate uncertainty - The student will troubleshoot and conduct research to contribute new knowledge about the unique phage. - The student will analyze data from the previous week and use them to make informed decisions on experiments for upcoming weeks |
| --- | --- |
| **How is it assessed:** | Graded lab notebooks of students due every week. Graded reflection assignments also due every week. |

Q. Reflect on your experience with failure as a part of authentic research and consider the following as you reflect: a. What have you learned from your failure? Provide details.

Sample student response: *During some of our direct isolations, we had a lot of contamination. However, for example, 3 weeks, we had 2 soil samples and did direct isolation on them for ABE 226 Lab Section: 11:30-1:30 4 a total of 4 plates, but only one plate out of all the samples was contaminated. There was much confusion on why this was contaminated. We discussed with the peer TA and she helped us with our aseptic technique, looking over our shoulder to ensure we were doing it correctly. Another failure we have had is not being able to find phages. Most of our samples come back entirely blank, with no plaques or contamination. We hope to find phages in our enriched isolation sample. We have learned that it is not easy to find something that is 1 million times, or even more, smaller than you.*

Q. Think of an experiment to perform if you continue to obtain more than one morphology in your phage preparations (e.g. some plaques are large and clear, and some are much smaller and cloudy).

a. How would you determine that an individual phage yielded two different morphologies?

Sample student response: *An experiment to perform if two different morphologies are found in phage preparations is called streaking, where lysogens are isolated away from exogenous infecting phage. This is a form of purification that ensures that only one type of phage remains present, meaning any future plaques formed by phages would be the same type of phage, regardless of morphology. Different morphologies after a few rounds of purification can hint at a phage that creates more than one type of morphology. Thus, this experiment should be carried out.*

b. What could be a possible mechanism by which one phage could yield two different morphologies?

Sample student response: *One phage may form plaques with different morphologies due to many factors, such as different differences in the smeg cultures used, differences in incubation times or temperatures, differences in the stressors that affect the phages that make each plaque, and differences in concentration of phages plated, such as in serial dilutions or uneven spreading of phages on a plate.*

______________________________________________________________________________

**ABET Student Outcome 7 - An ability to acquire and apply new knowledge as needed, using appropriate learning strategies.**

| **Course learning outcome**  Students will be involved in iteration | **Justification of mapping the course learning outcome to ABET student outcome 7**   - The student will troubleshoot and conduct research to contribute new knowledge about the unique phage. |
| --- | --- |
| **How is it assessed:** | Graded Reflection Assignments asking them to reflect on their failures and troubleshooting strategies to get success. Students also report their failures or use of alternative strategies to conduct their experiments in their Lab Notebooks. |

Q. Read this paper: Gallet et al. BMC Microbiology 2011, 11:181 (https://bmcmicrobiol.biomedcentral.com/articles/10.1186/1471-2180-11-181) and answer these questions:

a) How do you think the morphology of a plaque develops?

Sample student response: *It develops by a phage or a few phages infecting a bacterium and either following the lytic or lysogenic cycle. The phage then causes cell death radially from the first point of infection. The lytic cycle usually produces clear plaques and the lysogenic cycle produces hazy plaques. I believe that additional factors, such as high or low bacterial concentration, type of bacterial host, as well as phage concentration in one area, can affect the size, shape, and overall morphology of the plaques.*

Q. What is a web plate? Why do you think that a “web pattern” is critical for isolating high numbers of phage particles? What if your plate incubates too long and it becomes completely lysed? Should you use that plate for your titer? Why or why not?

Sample student response: *A web plate occurs when there are so many plaques, that they cover most of the plate and just the borders of the plaques touch, generating a “webbed” pattern. This pattern is important for isolate phage particles because there are many phage particles present (as evidenced by the large number of plaques on the plate) but not so many that there aren’t enough bacteria present to keep the phages reproducing and “alive”. This is what happens when it incubates too long and becomes completely lysed, therefore it cannot keep reproducing because there aren’t enough bacteria present and the phages begin to break down. That plate should not be used because the phage is not able to continue replicating and therefore using to for later activities wouldn’t result in any or possibly very few plaques.*

**LAB NOTEBOOK EVIDENCE**

**ABET Student Outcome 1 - An ability to identify, formulate, and solve complex engineering problems by applying principles of engineering, science, and mathematics**

| **Course learning outcome**  Students will be involved in the use of scientific practices. | **Justification of mapping the course learning outcome to ABET student outcome 1**   - The student will be able to explain the experimental basis of techniques used, indicating the significance of the work, presenting, calculating, and discussing the data, and drawing conclusions. - The student will be able to identify and define the basic terms within the field of biotechnology - The student will acquire basic research skills. such as the ability to perform techniques currently used in cell, molecular, and microbiology, while understanding the rationale behind the specific approaches. - The student will gain experience in dissecting and extracting pertinent information from scientific journal articles. - The students will propose solutions to troubleshoot and re-design or improve failed experiments. |
| --- | --- |
| **How is it assessed:** | **Graded Lab Notebooks and Reflection Assignments due every week.** |

Examples from students’ lab notebook:

*
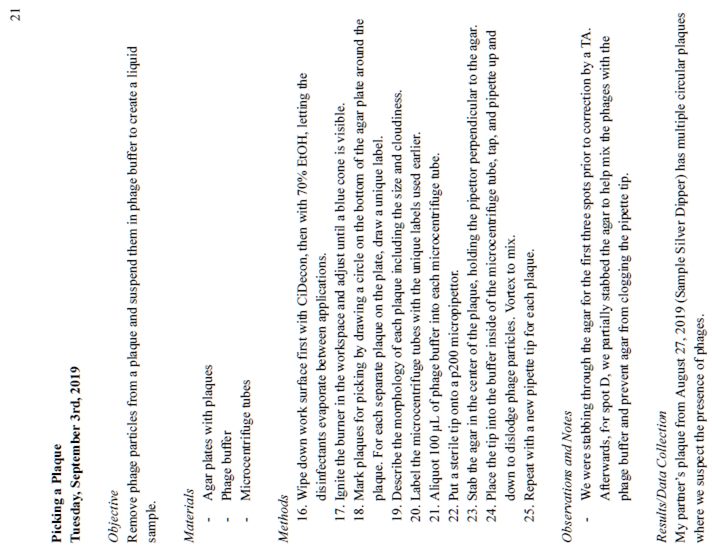
*

*
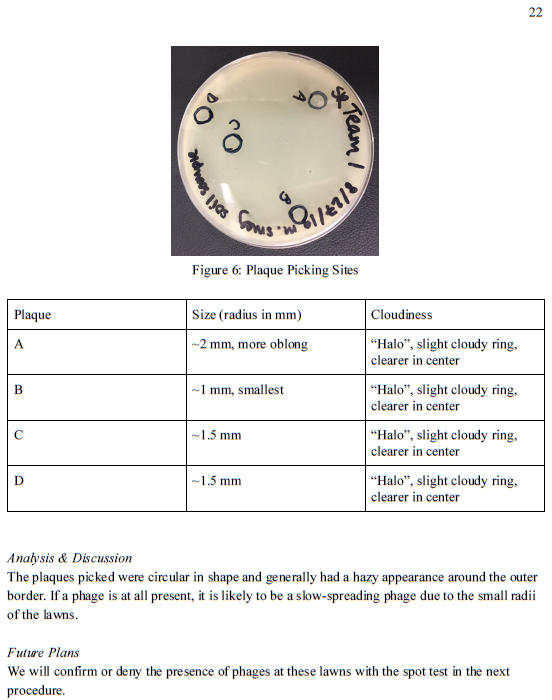
*

______________________________________________________________________________

**ABET Students Outcome 2 - An ability to apply engineering design to produce solutions that meet specified needs with consideration of public health, safety, and welfare, as well as global, cultural, social, environmental, and economic factors**.

| **Course learning outcome**  Students will be involved in broadly relevant or important work. | **Justification of mapping the course learning outcome to ABET student outcome 2**   - The students will isolate new phages by the end of the semester which has implications for public health as phages could provide a solution to antibiotic resistance - The student will compare the structure and properties of the phage to other phages - The student will share the information gathered with other students in the class and the scientific community outside the class |
| --- | --- |
| **How is it assessed:** | Graded Final Paper and Final Presentation due at the end of the semester. Graded Lab Notebooks and Reflection Assignments due every week. |
| **Course learning outcome**  Students will be involved in broadly relevant or important work. | **Justification of mapping the course learning outcome to ABET student outcome 2**   - The students will isolate new phages by the end of the semester which has implications for public health as phages could provide a solution to antibiotic resistance - The student will compare the structure and properties of the phage to other phages - The student will share the information gathered with other students in the class and the scientific community outside the class |
| **How is it assessed:** | Graded Final Paper and Final Presentation due at the end of the semester. Graded Lab Notebooks and Reflection Assignments due every week. |

Example from students’ lab notebook:


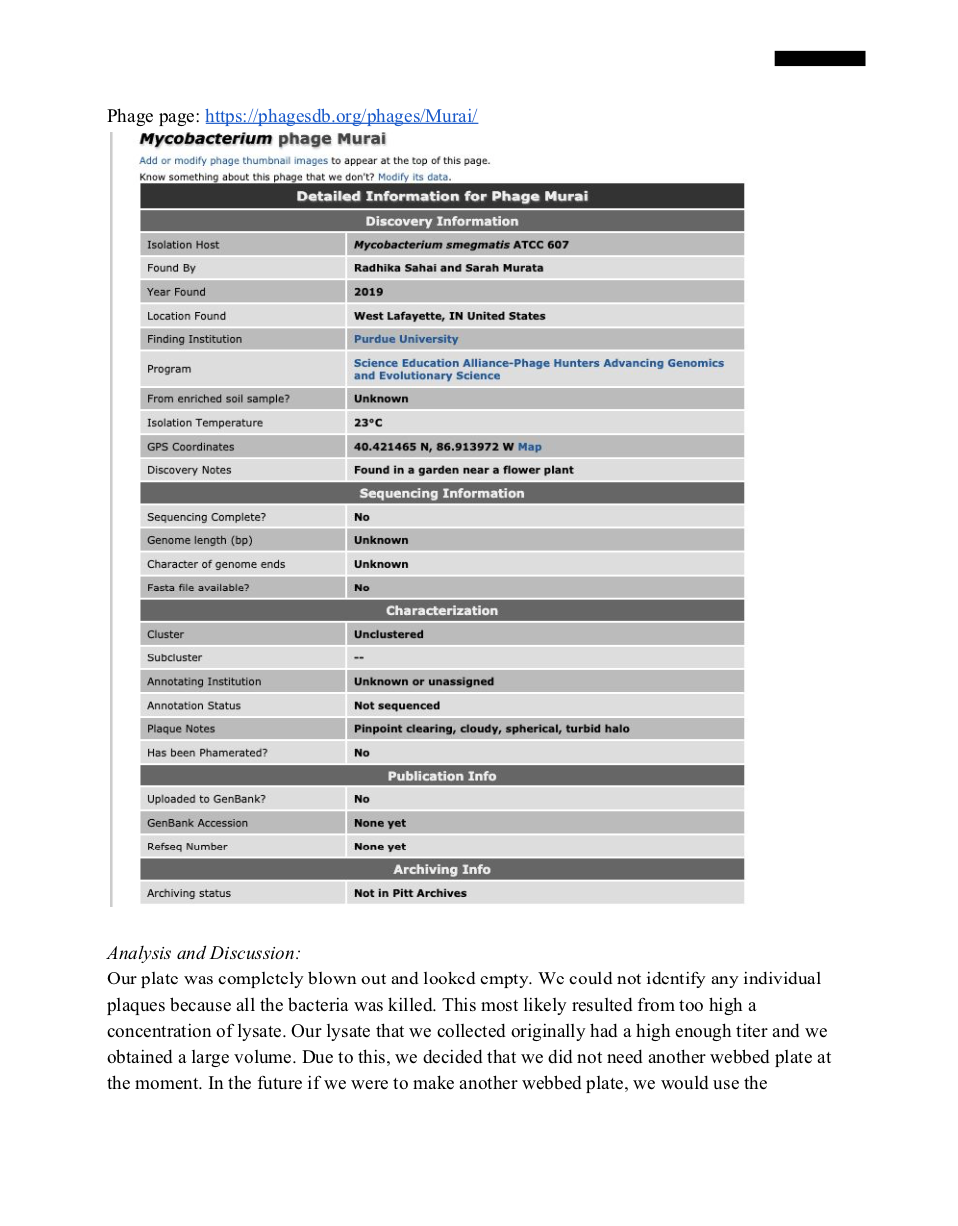


**Student Outcome 5 - An ability to function effectively on a team whose members together provide leadership, create a collaborative and inclusive environment, establish goals, plan tasks, and meet objectives.**

| **Course learning outcome :**  Students will be involved in collaboration | **Justification of mapping the course learning outcome to ABET student outcome 5**   - The student will work on a team and communicate their results - Students will be responsible for collaborating with others, dividing tasks, managing project materials and milestones, and making progress such that they meet all the goals by the end of the term |
| --- | --- |
| **How is it assessed:** | Graded Lab Notebooks due every week. Graded Final Paper and Final Presentation due at the end of the semester. |

Examples from students’ lab notebook:


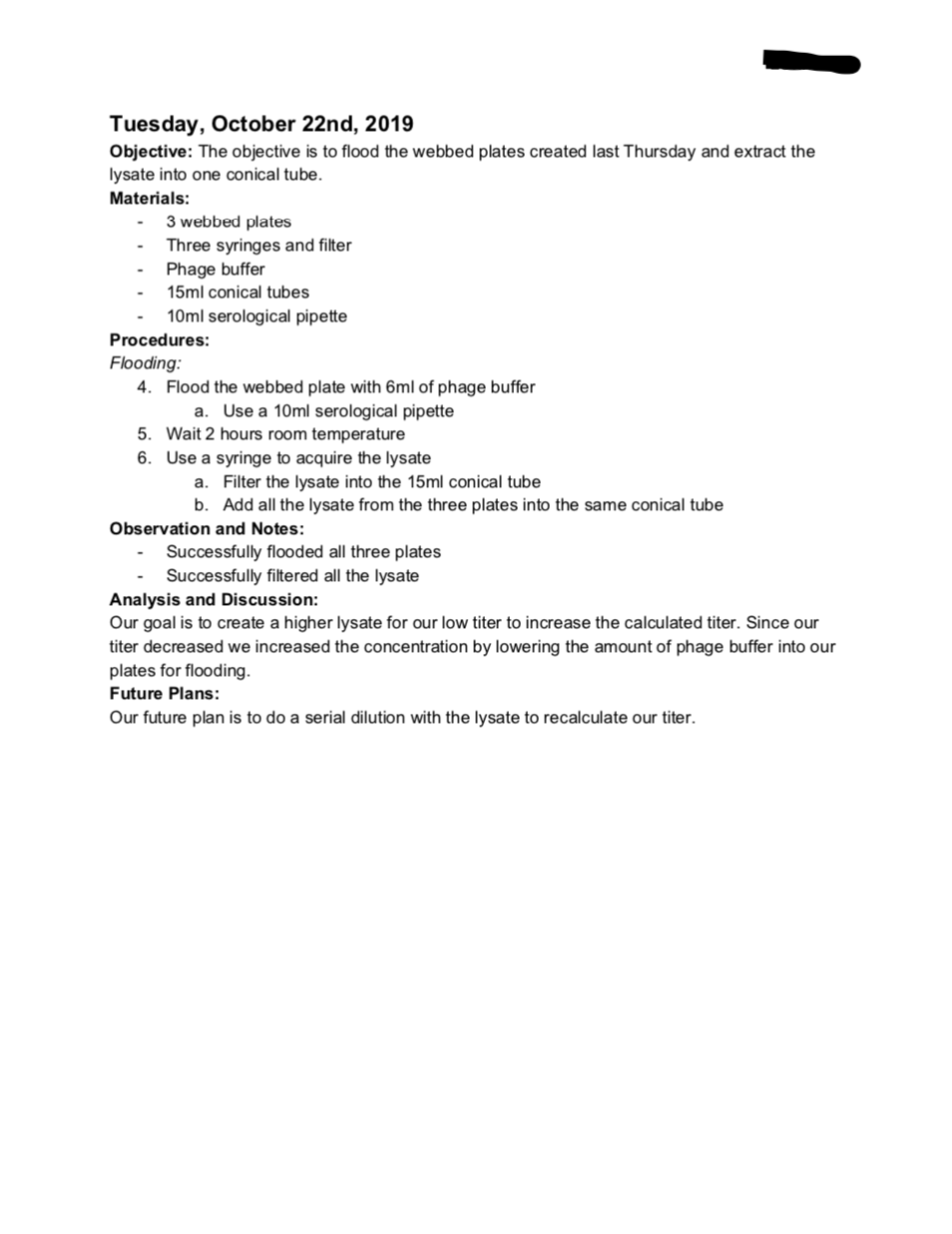


**Student Outcome 6 - An ability to develop and conduct appropriate experimentation, analyze and interpret data, and use engineering judgment to draw conclusions**

| **Course learning outcome**   - Students will be involved in the process of discovery - Students will be involved in iteration | **Justification of mapping the course learning outcome to ABET student outcome 6**   - The student will isolate and characterize a unique mycobacteriophage. - The student will be able to navigate uncertainty - The student will troubleshoot and conduct research to contribute new knowledge about the unique phage. - The student will analyze data from the previous week and use them to make informed decisions on experiments for upcoming weeks |
| --- | --- |
| **How is it assessed:** | Graded lab notebooks of students due every week. Graded reflection assignments also due every week. |

Examples from students’ lab notebook:


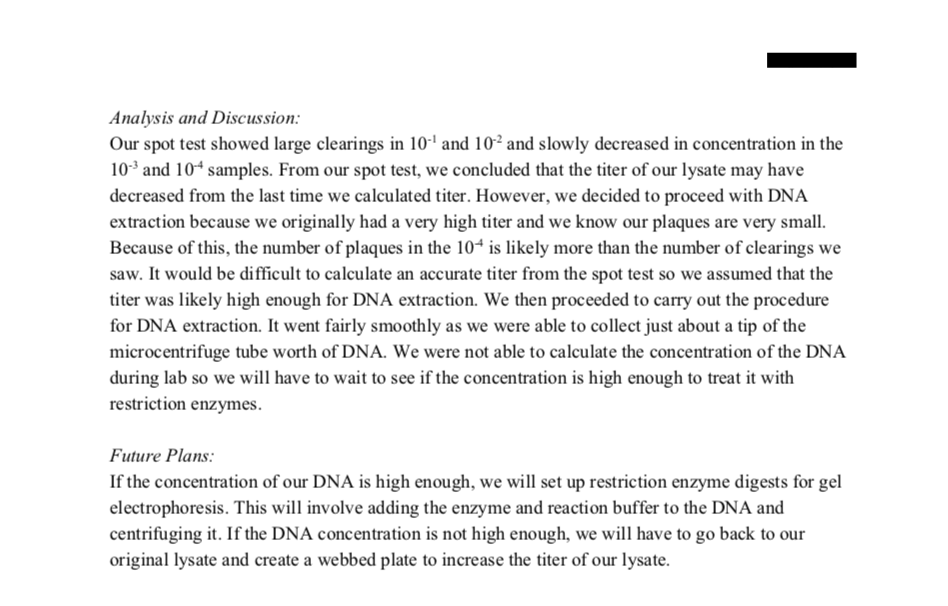


**Student Outcome 7 - An ability to acquire and apply new knowledge as needed, using appropriate learning strategies.**

| **Course learning outcome**  Students will be involved in iteration | **Justification of mapping the course learning outcome to ABET student outcome 7**   - The student will troubleshoot and conduct research to contribute new knowledge about the unique phage. |
| --- | --- |
| **How is it assessed:** | Graded Reflection Assignments asking them to reflect on their failures and troubleshooting strategies to get success. Students also report their failures or use of alternative strategies to conduct their experiments in their Lab Notebooks. |

Examples from students’ lab notebook:


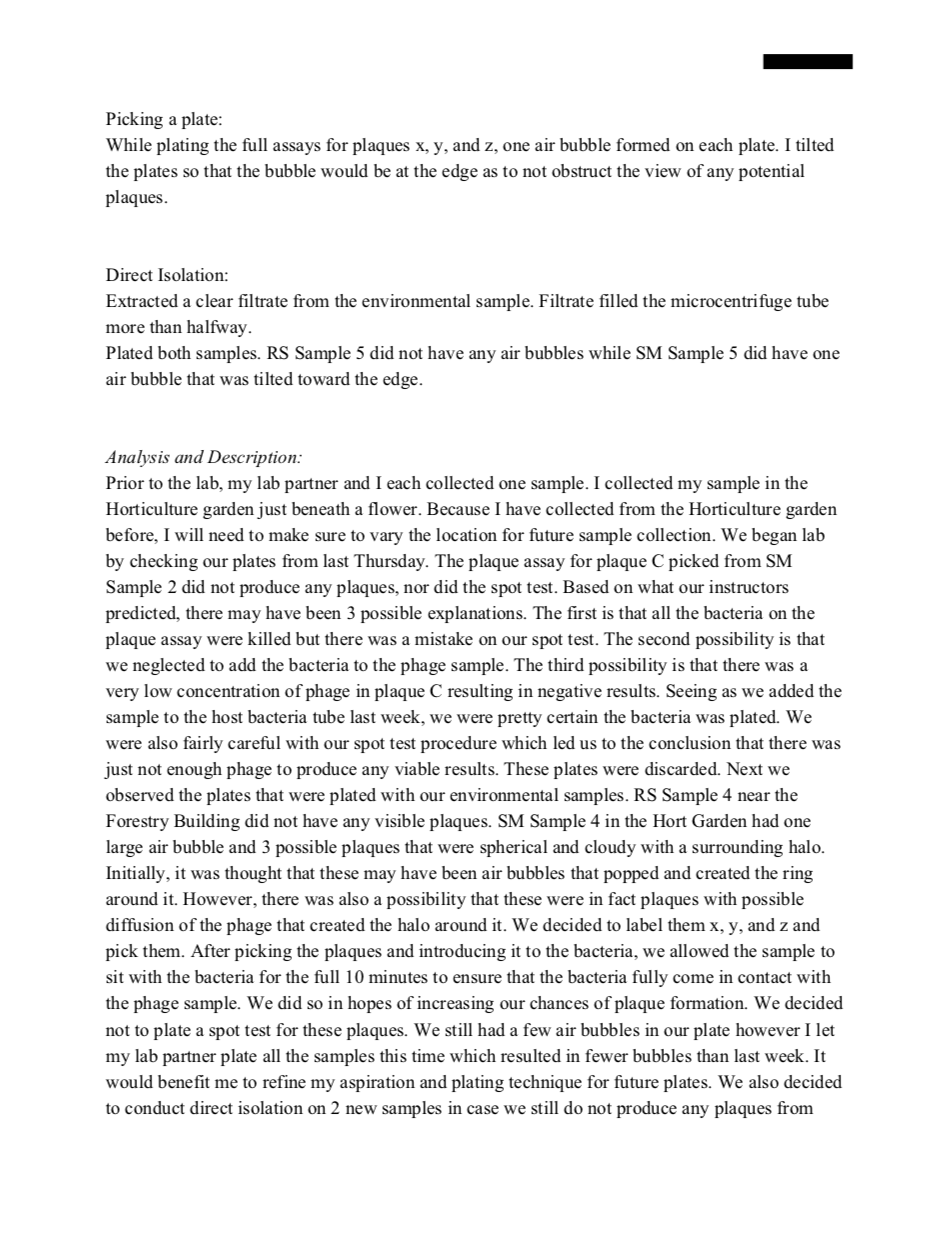


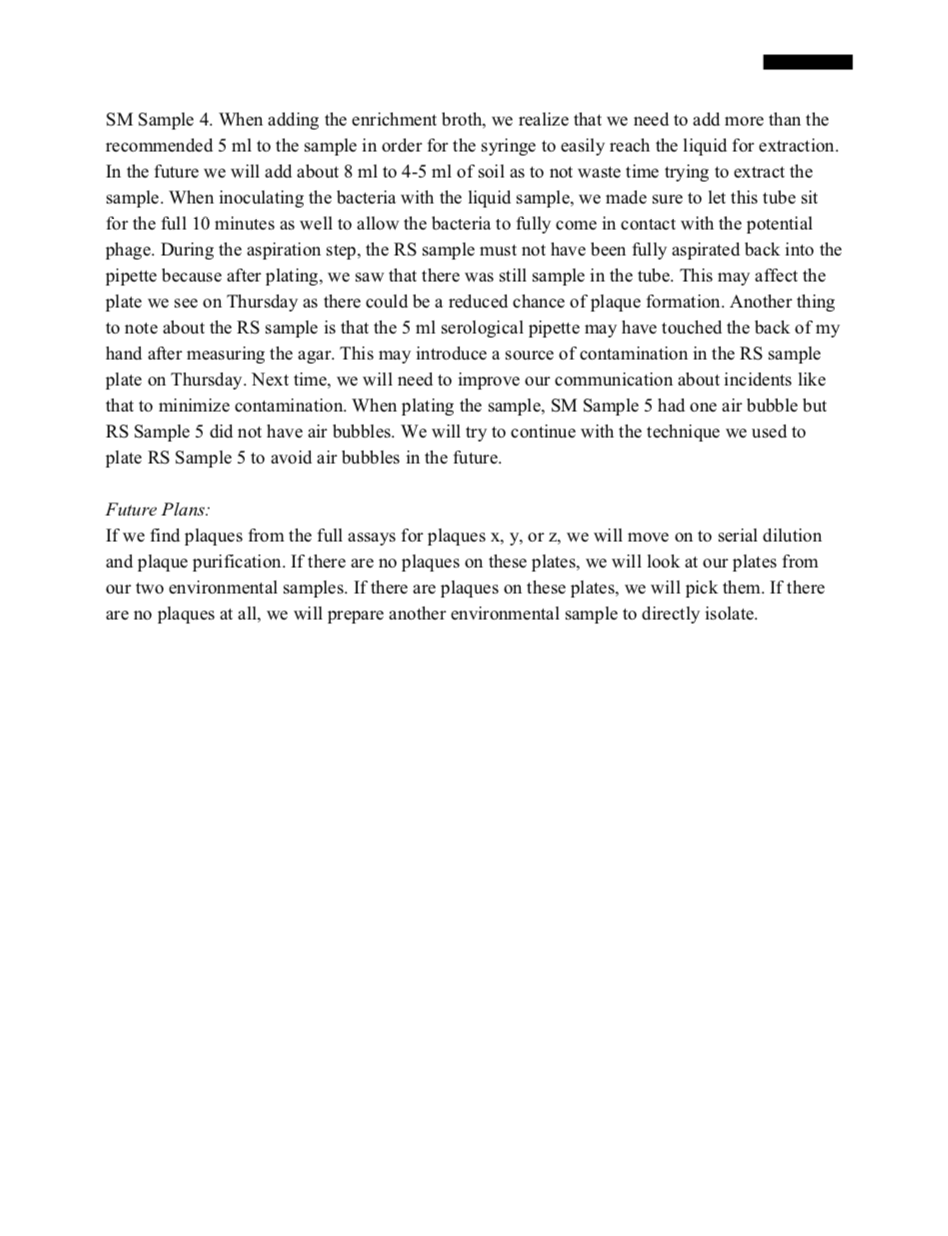

Supplement: Supplementary file 1 — APPENDIX S1: Examples from student reflections and lab notebooks. [file BMB-50-510-s001.docx]
